# Positional Obstructive Sleep Apnea: A Model for Precision Sleep Medicine

**DOI:** 10.3390/ohbm7020023

**Published:** 2026-06-28

**Authors:** Avneesh Prabakar, Shervin Eskandari, Tej Murudkar, Wenzhan Song, Xiaoyue Liu, Younghoon Kwon, William J. Healy

**Affiliations:** 1 Medical College of Georgia, Augusta University, Augusta, GA 30912, USA; 2 College of Engineering, University of Georgia, Athens, GA 30602, USA; 3 Rory Meyers College of Nursing, New York University, New York, NY 10010, USA; 4 Division of Cardiology, University of Washington, Seattle, WA 98195, USA; 5 Division of Pulmonary, Critical Care, and Sleep Medicine, Medical College of Georgia, Augusta University, Augusta, GA 30912, USA

**Keywords:** precision sleep medicine, positional obstructive sleep apnea, upper airway collapsibility, individualized therapy, clinical phenotyping

## Abstract

Positional obstructive sleep apnea (POSA) is a subtype of obstructive sleep apnea in which the apnea–hypopnea index (AHI) is significantly greater in the supine position than in non-supine positions. However, POSA remains underrecognized and lacks a universally accepted clinical definition. POSA’s prevalence can exceed 50% of OSA patients and occurs most frequently in patients with mild/moderate disease. In the supine position, gravity-dependent posterior displacement of the tongue and soft palate can increase upper airway collapsibility and drive the pathophysiology of POSA. These disturbances can carry meaningful cardiovascular consequences, including acute blood pressure surges that correlate with oxygen desaturation severity. As OSA is increasingly understood as a heterogeneous disorder shaped by variable anatomical and physiological traits, POSA offers a clinically actionable framework for precision sleep medicine. Current therapeutic strategies demonstrate that targeting the specific mechanisms of POSA can improve outcomes beyond what standardized CPAP treatment achieves alone. Racial and ethnic disparities in both POSA prevalence and treatment adherence further highlight the importance of individualized, culturally informed care. This review synthesizes the current understanding of the mechanisms, epidemiology, and therapeutic implications of POSA and evaluates its role as a model for individualized therapy in obstructive sleep apnea treatment.

## 1. Introduction

Obstructive sleep apnea (OSA) is conventionally understood as recurrent upper airway collapse during sleep, inducing sleep disturbances and decreased blood oxygenation [1]. Despite over 936 million men and women worldwide having some form of obstructive sleep apnea, the condition remains substantially underdiagnosed [2,3]. The systemic effects of OSA include cognitive dysfunction, hypertension, insulin resistance, and increased mortality, underscoring the importance of treatment [1].

OSA has traditionally been categorized and treated based on the apnea–hypopnea index (AHI). However, reliance on AHI as the primary, and perhaps sole, metric of OSA severity has been increasingly questioned, as it does not take into account symptom severity nor underlying mechanisms [4]. Although the first line of therapy for OSA continues to be continuous positive airway pressure (CPAP), poor therapy compliance has elicited the need for alternative therapies for OSA [5]. In addition, several cognitive deficits caused by OSA can only be partially treated by CPAP, illustrating that an overreliance on CPAP can be insufficient for OSA treatment [6].

In recent years, OSA has been viewed as a heterogeneous disease with considerable pathophysiological variability [7]. Many of the physiological traits that can be attributed to OSA, such as upper airway collapsibility and arousal threshold, vary widely across individuals with the disorder [8]. As a result, interest in personalized treatment for OSA has continued to increase. These strategies focus on identifying dominant physiological traits to guide individualized therapy instead of uniform CPAP treatments [9].

**Figure 1.**
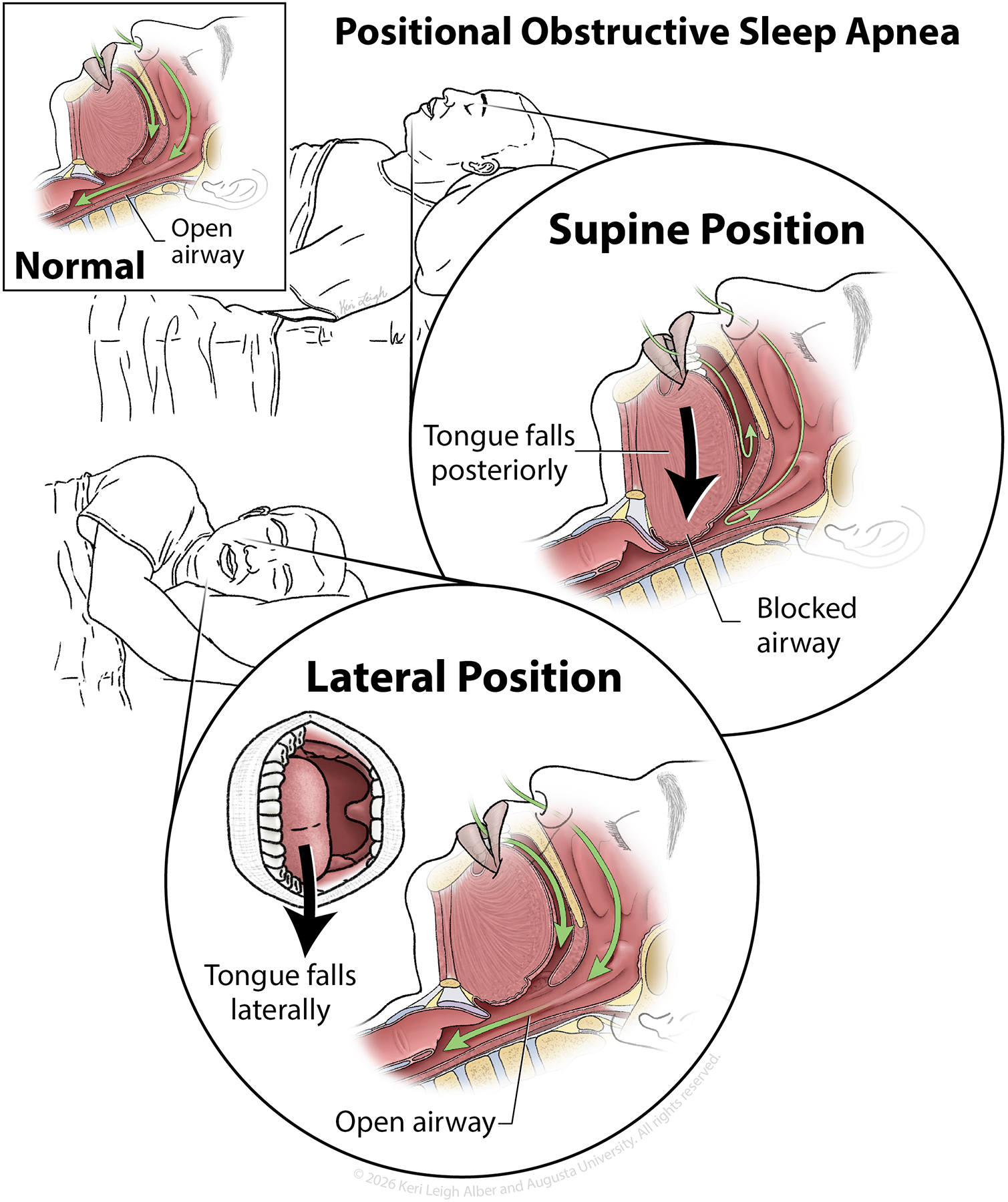
Illustration demonstrating positional obstructive sleep apnea (POSA). Inset (upper left) shows normal upper airway patency during sleep, with unobstructed airflow indicated by green arrows. In the supine position, gravity causes posterior displacement of the tongue against the posterior pharyngeal wall, resulting in airway obstruction. In the lateral decubitus position, the tongue falls laterally instead of posteriorly, preserving airway patency and airflow.

Positional obstructive sleep apnea (POSA), exhibited by over half of patients with OSA, represents a clinical subtype in which the apnea–hypopnea index (AHI) is significantly higher in the supine position compared to non-supine sleep [10]. In the supine position, upper airway resistance and tendency for the airway to collapse during sleep are greater [11]. Within this context, POSA can be characterized not only by positional differences in AHI but also as a gravity-dependent subtype defined by airway stability. Figure 1 illustrates the position-dependent airway stability differences in POSA patients. Although POSA is frequently considered a descriptive subtype of OSA, there may be meaningful physiological differences due to POSA’s positional dependence.

Moreover, POSA is modifiable. Since the severity of POSA is directly related to the amount of time spent sleeping in the supine position, positional therapy—techniques to deter patients from adopting the supine position—is currently used to treat POSA patients [12]. While this has demonstrated reductions in AHI for certain patients, making positional therapy a generalized treatment for all POSA patients proves limited in efficacy and adherence [13]. The numerous limitations of OSA treatments suggest the importance of looking into multimodal, patient-based treatment methods. POSA offers a unique opportunity in this regard since its severity depends on a single modifiable factor, sleep position. This positional dependency makes it a testable model for personalized therapy in sleep disorders.

This narrative review examines the pathophysiological mechanisms and therapeutic implications for positional obstructive sleep apnea. We explore how POSA can be used as a clinically implementable model for individualized therapy in OSA and the importance of precision sleep medicine.

## 2. Materials and Methods

### 2.1. Rationale

A narrative review was selected to integrate the epidemiology, mechanisms, and therapeutic implications of POSA while also situating it within precision sleep medicine. Unlike a systematic review, which requires a standardized format, a narrative review allows greater flexibility to engage in novel perspectives of the literature. The goal of this review is not to quantitatively pool effect sizes, but rather to position POSA as a clinically actionable phenotype of OSA.

### 2.2. Study Selection

This narrative review included original research studies, randomized controlled trials of positional therapy in OSA treatment, meta-analyses, systematic reviews, and foundational studies relating to the underlying mechanisms of positional effects.

Articles were included if they (1) examined positional obstructive sleep apnea or Positional Dependency in OSA, (2) investigated mechanisms related to positional variation, (3) evaluated positional therapy or alternative treatments for OSA, or (4) discussed endotyping or precision medicine approaches in OSA.

Articles were excluded if they (1) were non-English publications, (2) were not peer-reviewed, or (3) focused exclusively on centralized sleep apnea.

### 2.3. Search Strategy

A literature search was conducted using the PubMed/National Library of Medicine Online Database to identify articles fitting the inclusion criteria. Article selection began in March 2026. Searches were conducted using a combination of Medical Subject Headings (MeSH) and free-text words, and included studies published from 2004 to 2026. The following Boolean search strings were utilized:

1. (“obstructive sleep apnea” OR “OSA”) AND (“positional” OR “supine” OR “prevalence”) AND (“classification” OR “criteria” OR “definition”)
2. (“sleep apnea”) AND (“body position” OR “supine” OR “lateral”) AND (“collapsibility” OR “loop gain” OR “arousal threshold” OR “lung volume”)
3. (“positional obstructive sleep apnea” OR “POSA”) AND (“positional therapy” OR “wearable” OR “compliance” OR “oral appliance” OR “combination therapy”)
4. (“obstructive sleep apnea”) AND (“pharmacotherapy” OR “drug therapy” OR “weight loss” OR “GLP-1” OR “severity reduction”)
5. (“sleep apnea”) AND (“cardiovascular” OR “blood pressure surge” OR “hypoxic burden” OR “phenotype” OR “health disparities”)

From the results, titles and abstracts were screened for their relevance to the manuscript. Additional relevant articles were included with a manual review of reference lists from key publications. In total, 50 articles were included in the review.

## 3. Results

### 3.1. Definition of POSA

A major challenge in the study of positional obstructive sleep apnea (POSA) is the lack of a universal definition. Currently, the generally accepted definition for POSA is the Cartwright definition, characterized as the apnea–hypopnea index (AHI) being at least two times greater when sleeping in the supine position compared to a non-supine position [14]. While AHI quantifies the number of respiratory events per hour of sleep, it has been increasingly criticized for its inability to encompass the numerous clinical features of sleep, such as event duration and positional variability [4]. Furthermore, AHI does not take into account symptom severity for other non-supine positions. For example, an individual who exhibits significant apnea in a non-supine position can still meet the Cartwright criteria for POSA, even though apnea does not solely occur in the supine position—frequently termed “non-exclusive POSA” [15]. To address this limitation, numerous studies have used the term exclusive POSA (ePOSA). For example, Kang et al. defined ePOSA according to the criteria that non-supine AHI is normalized to an AHI less than or equal to 5 per hour [16]. Recent studies have also identified a lateral positional OSA phenotype, illustrating that positional dependency can extend beyond supine-related disease [17]. Importantly, these differing phenotypes have clinical relevance [15]. Common POSA treatment approaches, such as positional therapy, differ significantly in efficacy between non-exclusive POSA and ePOSA patients and will be discussed further in Therapeutic Implications. Therefore, solely relying on AHI thresholds may not adequately capture the heterogeneity in sleep apnea.

### 3.2. Prevalence and Age Factors

Positional obstructive sleep apnea (POSA) represents a significant portion of OSA patients, although the exact prevalence depends on the study population and diagnostic criteria. In a 2020 multicenter analysis, approximately 53.5% of OSA patients demonstrated positional OSA, with the prevalence increasing among patients with mild OSA and decreasing among patients with severe OSA [18]. These findings illustrate that positional dependence is particularly important during the earlier stages of disease. Furthermore, in-home polysomnography has confirmed the high prevalence of positional OSA among OSA patients, supporting that positional dependence is truly common in OSA and not simply attributable to the diagnostic methods used in sleep laboratories [19]. Age also plays an important role in POSA epidemiology. Studies focusing on OSA in elderly patients have shown that positional dependence remains prevalent even in older adults. For example, Iannella et al. found no significant difference in POSA prevalence between adult and elderly patients, even across three different classification systems [20]. This refutes the commonly held notion that positional dependence tends to decrease as age increases. Collectively, these studies highlight the significant prevalence of POSA across age groups, even despite diagnostic criteria differences.

### 3.3. Anatomical Mechanics

The anatomical mechanics of positional obstructive sleep apnea are largely dependent on the effects of gravity on upper airway anatomy during sleep. Upper airway collapsibility is determined by the interaction of craniofacial structures, such as the tongue, soft palate, and pharyngeal fat pads. When individuals have an elongated soft palate, fat accumulation in the pharyngeal fat pads, or a shortened mandible length, the propensity of the upper airway to become obstructed is greater [21]. OSA occurs when soft tissue narrows the airway, specifically at the velopharynx (the narrowest airway region). When individuals assume the supine position, gravitational forces promote posterior displacement of the tongue and soft palate, altering airway geometry and pressure inside the velopharynx [22]. These positional changes increase the likelihood of airway collapse, elucidating the POSA phenotype. Recent physiologic studies highlight that obstructive events in POSA are driven not only by craniofacial structure arrangement but also by physiological traits. The supine posture reduces lung volumes, such as functional residual capacity, decreasing caudal traction on the upper airway and increasing the likelihood of collapse [23]. The integration of these anatomical and non-anatomical traits in relation to modern endotyping frameworks will be further explored in Section 5.3. Ultimately, these anatomical and biomechanical factors help explain airway obstructions in individuals with OSA and why these respiratory disturbances predominantly occur during supine sleep.

### 3.4. Cardiovascular Outcomes

The acute cardiovascular consequences of obstructive sleep apnea are mediated by the physiological responses that occur with each apneic episode, and these responses are highly relevant to positional obstructive sleep apnea (POSA). Every episode triggers a transient rise in blood pressure during and immediately following the episode. Kwon et al. directly captured these surges using a continuous beat-to-beat blood pressure monitoring system that is integrated into polysomnography. These mean systolic pressure surges associated with respiratory events ranged from 5 to 19 mmHg, though these surges were highly heterogeneous both between and within patients [24]. They further identified a linear correlation between the degree of oxygen desaturation and the magnitude of blood pressure surge, with significant surges (≥10 mmHg) occurring more frequently during events with more severe desaturation (≥5%). Since the supine position increases upper airway collapsibility and tends to be associated with greater oxygen desaturations, it is hypothesized that the supine position may subject the patient to greater acute cardiovascular stress [25].

However, it is important to note that long-term epidemiological data indicate the POSA phenotype has a lower baseline risk for cardiovascular diseases, including atrial fibrillation and heart failure, compared to non-positional OSA populations [16]. The lower long-term risk of cardiovascular disease can be explained by the fact that POSA cohorts often present with lower average BMIs and baseline AHIs compared to non-positional cohorts [15]. Therefore, the acute mechanism of the supine position provoking cardiovascular surges should be decoupled from longitudinal risk.

Nevertheless, these effects are not distributed equally across populations. Healy et al. noted that although African American and white adults exhibit similar overall OSA prevalence, African Americans tend to have more severe disease and approximately twice the odds of resistant hypertension when OSA is present. This disparity likely reflects both biological susceptibility and structural barriers to care [26].

### 3.5. POSA as a Distinct Phenotype

Although POSA is widely treated as a positional variant of OSA, it may represent a distinct clinical phenotype within the broad spectrum of obstructive sleep apnea. OSA has been increasingly identified as a heterogeneous disorder driven by multiple anatomical and physiological mechanisms rather than a single pathophysiology. Eckert et al. studied the interaction of numerous physiologic traits in OSA patients, including upper airway collapsibility, upper airway dilator muscle responsiveness, respiratory arousal threshold, and ventilatory control system sensitivity. They discovered substantial variability in these traits between OSA patients, evidencing that OSA is not a “one-size-fits-all” disorder [8]. Rosales et al. investigated subgrouping OSA patients by symptom-based phenotypes, finding that sorting OSA patients into clinical profiles can allow for more effective targeted interventions and optimize therapeutic outcomes [27]. Furthermore, these differences in physiologic traits give rise to distinct clinical phenotypes that vary in symptom severity and treatment response. In this framework, positional dependence can serve as a modifier of airway mechanics, as the supine position can increase the likelihood of these obstructive events. POSA patients consistently report a milder disease severity and may be better suited for positional therapy rather than standardized CPAP treatment, illustrating that POSA can be viewed as a clinically actionable subtype of OSA [28]. The recognition of POSA as a distinct phenotype may therefore facilitate individualized therapies and improve treatment selection in obstructive sleep apnea.

## 4. Therapeutic Implications

### 4.1. Positional Therapy

Positional therapy is an intervention strategy that aims to prevent POSA patients from adopting the supine position during sleep. In the past, challenges prevented the evaluation of the efficacy of positional therapy, making it difficult to implement across wide patient populations. In addition, long-term therapy compliance with traditional positional therapies can be poor. Bignold et al. reported that less than 10% of study patients continued tennis-ball technique positional therapy, in which a tennis ball is fastened to the back of the patient to discourage supine sleep, over 30 months [29]. However, technological advancements have renewed interest in positional therapy in hopes of increasing patient satisfaction and adherence. Modern sleep positioners attempt to mitigate discomfort by utilizing low-profile wearables, which can deliver gentle vibrations when a certain supine angle is reached to encourage a subconscious shift out of the supine position [30]. Ravesloot et al. investigated next-generation vibrotactile devices in the treatment of POSA and demonstrated high compliance under the study conditions, but a lack of reliable long-term adherence data creates a barrier for clinical implementation [12]. Numerous studies indicate that positional therapy can substantially reduce the AHI in patients with select patients—although long-term compliance is heavily debated—in select cohorts with mild-to-moderate OSA [5]. Consequently, positional therapy may be more effective in patients in whom anatomical airway collapsibility occurs in the supine position. Permut et al. indicated that positional therapy can preferentially benefit POSA patients and provide better long-term compliance compared to CPAP, supporting the usage of positional therapy for specific subsets of OSA patients [31]. From a comparative effectiveness standpoint, some populations demonstrate higher satisfaction and adherence to certain positional therapy devices, while positive airway pressure is generally more effective at reducing AHI across general populations [32]. Individuals also exhibit substantial night-to-night variability in supine sleep time, meaning that positional therapy may not have the same effects under highly variable home sleep environments [33].

As discussed previously, treatment efficacy differs between ePOSA and non-exclusive POSA patients. In ePOSA patients, where gravity-dependent airway collapse is the driving factor behind respiratory events, sleep-positioning treatment devices can be highly effective [32]. On the other hand, meta-analyses reveal that positional therapy fails to yield significant improvements in AHI or oxygen saturation for non-exclusive patients, necessitating multimodal therapy [34]. While the long-term effectiveness of modern positional therapy techniques needs to be more extensively studied, it nonetheless underscores the importance of identifying POSA phenotypes in individual patients.

### 4.2. Multimodal Care

Treatment of positional obstructive sleep apnea (POSA) can require a multimodal approach integrating different therapies to effectively address the numerous anatomical and physiological factors of the disease. Obesity is correlated with OSA, and weight loss is regularly recommended as a behavioral intervention, as this can reduce pharyngeal fat deposition constricting the upper airway [19]. Consequently, weight loss can relieve some of the symptoms experienced by patients with POSA. In addition to lifestyle interventions, oral appliance therapy can serve as an adjunctive or alternative therapy for patients with POSA unable to tolerate CPAP. Mandibular advancement appliances, one of the most common oral appliance therapies, function by moving the mandible and tongue anteriorly, enlarging the upper airway and decreasing the chance of collapse [35]. Kim et al. indicated that these oral appliances have significant benefits on cardiovascular outcomes and other symptoms of OSA, illustrating their effectiveness for patients [36]. In fact, Qiao et al. directly compared the effectiveness of positional therapy and oral appliance therapy for patients with POSA, reporting that oral appliance therapy resulted in a greater reduction in AHI than positional therapy [37]. These results highlight the potential of oral appliance devices as an efficient and effective treatment for POSA. Moreover, combination strategies may provide additional benefits for POSA patients compared to using individual treatments themselves. Dieljtens et al. assessed the usage of a sleep position trainer (positional therapy) in conjunction with mandibular advancement devices for patients with POSA, and found that this combination therapy yielded a higher therapeutic efficiency than a single treatment alone [38]. The added effectiveness of combination therapy may reflect the multifactorial nature of positional obstructive sleep apnea, highlighting the potential of various non-CPAP treatments in the management of the disease.

### 4.3. Pharmacotherapy

Apart from the established treatments for positional obstructive sleep apnea (POSA), numerous pharmacologic treatments have emerged as supplemental therapies that target the underlying drivers of obstructive sleep apnea. Glucagon-like peptide-1 (GLP-1) receptor agonists have gained recent traction for their effects on weight loss and cardiovascular health. While originally developed for diabetes treatment, these drugs promote significant weight loss and are associated with significant reductions in AHI in obese patients [39]. Mechanistically, the weight loss promoted by these GLP-1 receptor agonists can decrease fat deposition in pharyngeal soft tissue and prevent upper airway narrowing, thereby indirectly modifying the anatomical risk factors of POSA. One drug in particular, Tirzepatide, showed significant improvements in AHI and events per hour for OSA patients in a clinical trial, but further research is needed to investigate the effects of these GLP-1 receptor agonists in POSA patients specifically [40]. In contrast, experimental pharmacotherapies targeting the non-anatomical aspects of OSA are currently being investigated. Combination therapy using noradrenergic–muscarinic agents, such as Atomoxetine and Oxybutynin, exhibited significant reductions in AHI over a short-term period [41]. However, these findings are derived from general OSA populations and cannot be readily translated to the POSA subtype. Although focused studies are needed to confirm the efficacy of these treatments for POSA-specific cohorts, these findings nevertheless highlight new directions in the development of pharmacotherapies for sleep apnea treatment.

### 4.4. Health Disparities and Beyond AHI

Racial and ethnic differences play an underappreciated role in the therapeutic implications of individuals with positional obstructive sleep apnea (POSA). Between different racial groups, prevalences in OSA widely differ, with one study reporting that African Americans exhibit higher severity of obstructive sleep apnea compared to their white counterparts [42]. This may be due to greater rates of obesity or could suggest that genetic factors play a role in susceptibility to sleep apnea. However, in a significant multicenter study, Chinese Americans demonstrated the highest prevalence of supine positional OSA, while African Americans exhibited the lowest prevalence of the supine phenotype [43]. This suggests that POSA prevalence in different racial and ethnic groups may be independent from OSA prevalence itself, potentially due to non-anatomical factors. For example, one sleep study demonstrated that young black males exhibit a significantly lower loop gain compared to age-matched and BMI-matched white males, suggesting that non-anatomical mechanisms can have a varying effect between patient populations [44]. Whether these physiological differences contribute directly to positional dependence is still unclear, but they illustrate the variance in traits across populations. Furthermore, treatment adherence for POSA and barriers to healthcare vary widely across different socioeconomic groups. In black populations, reported adherence to CPAP treatment is substantially lower compared to white patients, and these differences are further exacerbated by diagnostic tool limitations and healthcare coverage policies [43]. Rigid coverage policies, such as the requirement of 4 h of CPAP use at least 70% of nights, can disproportionately affect minority patients who have shorter sleep times and unstable work schedules [43]. These inequalities intersect with cultural beliefs and historical mistrust, as multiple studies have found that African Americans exhibit differences in beliefs regarding sleep [44]. Collectively, this indicates the importance of individualized and culturally informed treatments for positional obstructive sleep apnea.

Apart from these racial and ethnic differences, the apnea–hypopnea index (AHI) is currently used to evaluate treatment efficacy in OSA patients. To compensate for the limited utility of AHI, other methods such as diagnostic therapy and integrating clinical characteristics in OSA severity may better characterize POSA and provide new treatment paradigms [4].

## 5. Conclusions and Future Directions

### 5.1. Phenotype Recognition

The identification of positional obstructive sleep apnea (POSA) as a clinically distinct phenotype is a foundational step to identifying patient profiles and providing targeted therapies, and fundamental for advancing precision sleep medicine. With an approximate prevalence of 20–75% among OSA patients, POSA represents a significant subset of the OSA population and warrants dedicated attention [28]. Although the “one-size-fits-all” model of CPAP treatment for OSA patients provides standardized therapy, identifying supine-dependent individuals may help deliver tailored treatments and improve overall clinical outcomes.

### 5.2. Resolving Inconsistencies

Finding a standard clinical definition for POSA will likely require resolving the definition inconsistencies attributed to the disease. The Cartwright criteria, though historically relevant, does not account for the actual time spent sleeping in each position and symptom severity. To address these limitations, advanced classification frameworks have emerged, most notably the Amsterdam Positional OSA Classification (APOC). Instead of on time spent sleeping supine, the APOC focuses on the patient’s best sleeping position (BSP) versus worst sleeping position (WSP) [45]. The APOC framework systematically categorizes into three categories—APOC I (AHI < 5 in BSP), APOC II (AHI > 5 in BSP but lower than WSP), and APOC III (overall AHI > 40 and overall 25% reduction in BSP)—progressing from exclusive positional dependency to severe OSA, regardless of the position [46]. The APOC framework can be used to clinically diagnose patients with POSA and help implement phenotype-guided treatment strategies [46]. For example, APOC I individuals may receive pure positional therapy, whereas APOC II and III patients may receive multimodal treatment. Despite these new frameworks, there remains a lack of a generally accepted definition for POSA [45]. Addressing the variability in positional dependency between patients may help phenotypic classification and allow for individualized therapy.

### 5.3. Mechanism Integration

Despite ongoing research, the mechanisms underlying POSA remain incompletely characterized. While gravity-dependent airway collapse has been identified as a major mechanism elucidating the POSA phenotype, current models suggest it may not fully account for underlying physiological traits that contribute to disease expression. The integration of non-anatomical traits can provide a more comprehensive understanding of POSA. Modern physiological endotyping frameworks conceptualize OSA as the interaction between upper airway collapsibility, loop gain, arousal threshold, and upper airway muscle responsiveness [8]. Mechanistically, the supine position causes a decrease in functional residual capacity, increasing upper airway collapsibility and potentially contributing to ventilatory instability for POSA patients [22]. Simultaneously, a POSA patient exhibiting a low respiratory arousal threshold may awaken before the obstruction is cleared. Identifying whether a patient’s positional dependence is entirely driven by one of those factors or a combination of them can provide a clinical roadmap for treatment. Integration of these traits can allow clinicians to distinguish patients whose positional dependence is primarily anatomically driven from those with significant non-anatomical contributors. Further understanding the mechanisms of POSA and distinguishing phenotypes between patient populations can help inform more precise therapies.

### 5.4. Quality Measures

Current treatment paradigms for OSA, including POSA, are heavily reliant on AHI reductions as the primary measure of therapeutic success. However, data indicate that a reduction in AHI alone does not fully capture the multifactorial impact of the disease, nor does it consistently correlate with clinical improvement [47]. Future research should consider prioritizing multidimensional outcome measures, including sleep quality, daytime functioning, cognitive performance, cardiovascular risk, and patient-reported outcomes. Self-administered questionnaires, such as the Epworth Sleepiness Scale (ESS) and the Functional Outcomes of Sleep Questionnaire (FOSQ), can be routinely utilized to assess daytime sleepiness and functional impairment [48]. Additionally, disease-related quality of life outcomes can be assessed using tools, such as the Medical Outcomes Study 36-item shortform health survey (SF-36) [49]. For prognostic ability, Hypoxic Burden (HB) has emerged as a superior predictor of cardiovascular morbidity for OSA patients, and may also be implemented for POSA populations; however, its predictive validity remains to be verified for POSA cohorts [50]. These tools have their own limitations, and longitudinal studies are needed to evaluate the sustained effects of therapy on these clinically meaningful endpoints. However, moving beyond short-term AHI reduction toward a more comprehensive assessment of health outcomes will be essential in optimizing treatment strategies.

### 5.5. Phenotype-Guided Management and Precision Sleep Medicine

Precision medicine seeks to provide individualized therapy to patients based on their differing characteristics. The recognition of POSA has important implications for the progression of precision sleep medicine. Unlike other traits of OSA, which may be difficult to assess, POSA provides an actionable model because positional dependence is readily measurable and a targetable feature, placing it in a unique position for the advancement of sleep medicine.

The evidence reviewed suggests that a better understanding of positional dependence may help explain variability in treatment response, moving beyond the limitations of AHI alone. At the same time, more evidence is needed to support phenotype-guided management for POSA patients. Few studies have investigated the outcomes of identifying positional phenotypes, and the lack of a generally accepted POSA definition can limit comparison between studies and overall generalizability.

However, these limitations do not diminish the potential of POSA as a pathway to precision sleep medicine. Instead, it underscores the importance of new studies that investigate the utility of positional dependence as a decision-making tool. POSA represents one of the most actionable OSA phenotypes available, and the transition towards phenotype recognition to individualized therapy will require stronger evidence that phenotype-guided individualized therapy improves outcomes compared to traditional methods.

Ultimately, the investigation of positional dependence in obstructive sleep apnea highlights the potential of shifting towards patient-based treatment models and individualized therapy for sleep disorders.

## Data Availability

No new data were created or analyzed in this study. Data sharing is not applicable to this article.

## Abbreviations

The following abbreviations are used in this manuscript:

POSA: Positional Obstructive Sleep Apnea
OSA: Obstructive Sleep Apnea
AHI: Apnea–Hypopnea Index
